# Hypercrosslinked waste polycarbonate to remove heavy metal contaminants from wastewater

**DOI:** 10.1038/s41598-024-54430-0

**Published:** 2024-02-27

**Authors:** Hadiseh Masoumi, Ahad Ghaemi

**Affiliations:** School of Chemical, Petroleum and Gas Engineering, Iran University of Science and Technology, Tehran, Iran

**Keywords:** Hypercrosslinked polymer, Waste polycarbonate, Metal ions, Porous material, Environmental chemistry, Environmental impact, Chemical engineering

## Abstract

In this research, the waste polycarbonate was hypercrosslinked during the Friedel–Crafts reaction to eliminate metallic ions from the wastewater solution. The experiments for inspecting the adsorption behavior of lead and cadmium ions were conducted at the initial concentration of 20–100 mg/L, contact time of 10–80 min, temperature of 20–80 °C, and pH of 6–11. The isotherm, kinetic, and thermodynamic models have been used to explain the behavior of the metal ions removal process. The correlation coefficient and adsorption capacity of the kinetic model for cadmium ion have obtained 0.995 and 160.183 mg/g, respectively, and the correlation coefficient and adsorption capacity of the kinetic model for lead ion has obtained 0.998 and 160.53 mg/g, respectively, which declared that the cascade was not monolayer. The correlation coefficient of the Freundlich is calculated at 0.995 and 0.998 for Cd and Pb, respectively, indicating the resin plane was not homogenized. The n constant for cadmium and lead ions has been calculated at 2.060 and 1.836, respectively, confirming that the resin is not homogenized, and the process has performed well. Afterward, the values of enthalpy and Gibbs free energy changes were obtained at − 7.68 kJ/mol and − 0.0231 kJ/mol.K for lead ions, respectively, which implies the exothermic and spontaneous state of the process. The values of enthalpy and Gibbs free energy changes have been obtained at − 6.62 kJ/mol and − 0.0204 kJ/mol.K for cadmium ions, respectively, which implies the exothermic and spontaneous nature of the adsorption. Also, the optimal empirical conditions for lead and cadmium ions have been found at a time of 60 min, temperature of 20 °C, initial concentration of 100 mg/L, and pH of 10. At a time of 45 min, the diffusion coefficient and mass transfer coefficient for lead ions have been calculated at 0.1269 × 10^20^ m^2^/s and 0.2028 × 10^15^ m/s, respectively. In addition, at a time of 45 min, the diffusion coefficient and mass transfer coefficient for cadmium ions have been calculated at 0.1463 × 10^20^ m^2^/s and 0.1054 × 10^15^ m/s, respectively. Moreover, the mechanism study explains that the C–O–C and C–H in the aromatic groups have a crucial aspect in the bond formation among metallic ions and resin.

## Introduction

Water is an essential agent in both domestic and industrial cases^1^, this material is vital for the living of inhabitants on the earth^2^. Only a very small amount of water (0.3%) on the earth is applicable for drinking and other human use. Hence, preventing the pollution of water by organic and inorganic materials is crucial for preserving its quality^3^. Metallic ions especially lead, cadmium, and mercury have been recognized as hazardous contaminants in the water because of not being biodegradable, which causes severe damage to the human organism including cancer, respiratory harm, and kidney failure, thus, decreasing the concentration of metallic ions in the allowable content is required^4,5^. Heavy metal ions can deteriorate the water quality, threaten human and animal health, and damage the ecosystem balance and economic development^6^. For example, lead ion causes damage to the nervous system, reproductive system, kidney, liver, and brain^7^. Nickel ions can damage to nose and bone as well as lung cancer^6^. In addition, hexavalent chromium ion is very mutagenic^8^. The sources of metallic ions' production include battery production, petrochemical industries, electroplating, etc. There are various techniques for the removal of metallic ions such as chemical precipitation, electrochemistry, liquid–liquid extraction, microbial methods, and adsorption^9^. Each of the mentioned techniques has disadvantages, but adsorption is the most appropriate method owing to its versatility, low cost, without generating secondary pollution, and ease of use. In the adsorption process, adsorbent selection with the desired uptake capacity and reusability is a main aspect^10,11^. Different adsorbents have been employed including waste materials, microorganisms, activated carbon, and polymers. Each of these adsorbents has its benefits. For example, activated carbon is low-cost and high-yield adsorbent^8^, and poly(thiourea imine) is new with great potential in the removal of lead, copper, and cadmium ions^7^. In the newly published literature, it can be elucidated that polymeric materials have been extensively applied because of their favorable characteristics such as good mechanical and chemical stability, high efficiency, and recyclability. Nowadays, researchers focus on the aliphatic polycarbonates of the waste materials due to their ease of decomposition and ecofriendly^12^. In combination with their ready hydrolysis and low toxicity, aliphatic polycarbonates are attractive materials not only in agricultural or packaging applications but are also one of the most often used synthetic materials in (bio) medical and pharmaceutical fields as gene carriers^13^, drug delivery systems based on either nanoparticle, microspheres^14^ or hydrogels^15^. Moreover, hypercrosslinking of polymers is a good approach for promoting the uptake rate. The hyper-cross-linking process occurs with the help of the Friedel–Crafts reaction^16^. Generally, the hypercrosslinking process can increase the surface area of the polymers. Hyper-cross-linking polymers are typically fabricated from lightweight and low-price components and have suitable scalability. Additionally, the mentioned resins display desired stability to the temperature and chemical modifications^17^. Many scientists worked on the removal of heavy metal ions such as lead, cadmium, selenium, and etc. using various adsorbents, especially porous magnetic nanocomposites, and modified carbon nanotubes^18,19^. The list of hypercrosslinked polymers (HCPs) for the elimination of metallic ions is presented in Table 1. The results indicated that the applied polymeric adsorbents have a higher adsorption capacity to compare the other types of adsorbents. In addition, different kinds of isotherm and kinetic models were used to identify the behavior of the polymeric absorbents.

**Table 1:** Researches on the HCPs adsorbent for heavy metal ions.

Researcher	Adsorbent	Metal ions	T (°C)	C (ppm)	pH	a (m^2^/g)	q (mg/g)	Models			Ref
								Isotherm	Kinetic	Thermodynamic	
Nejad et al	Cyclodextrin polymer	Pb, Cd	25	250	7	–	285.126, 126.58	Langmuir, Freundlich, Temkin	–	–	^22^
Cheng et al	Crosslinked NDWJN2	Cu, Ni	30	–	–	580.00	116.28, 126.58	Langmuir, Freundlich	–	–	^23^
Sezgin et al	Polymeric acid hydrogel	Cu, Ni , Zn, Cr	20	300	2	–	3.10, 1.64, 6.43	Langmuir, Freundlich	Pseudo-First-Order, Pseudo-Second-Order	ΔH, ΔS, ΔG	^24^
Yang et al	HCPs resin chemically modified with thiourea	Pb, Cd, Cu	21	–	6	211.21	689.65, 432.90, 290.69	Langmuir, Freundlich, D–R	Pseudo-First-Order, Pseudo-Second-Order	–	^25^
Daminova et al	HCPs adsorbent	Au, Pt	25	60	8	792.00	1.05, 0.84	–	Pseudo-First-Order, Pseudo-Second-Order, Intra-Particle-Diffusion	–	^26^
James et al	Sulfonated HCPs	Sr, Cs	20	500	7	580.00	95.6, 273	Langmuir, D–R	Pseudo-First-Order, Pseudo-Second-Order	–	^27^
Ivanets et al	Hydroxyapatite	Cd, Co, Fe, Ni, Pb, Zn	25	40	4.5	240.00	0.12, 0.002, 0.13, 0.08, 0.072, 0.148, 0.01	–	Pseudo-First-Order, Pseudo-Second-Order	–	^28^
Masoumi et al	Hypercrosslinked Polystyrene	Cd	25	120	7	853.89	950.00	Langmuir, Freundlich, D–R Temkin	Pseudo-First-Order, Pseudo-Second-Order, Elovich, Fractional order	ΔH, ΔS, ΔG	^29^
Masoumi et al	Hypercrosslinked Polystyrene	Pb, Ni	20	60	10	853.89	174.00, 116.67	Langmuir, Freundlich, Redlich-Peterson, Temkin	–	ΔH, ΔS, ΔG	^29^
Masoumi et al	Hypercrosslinked Polystyrene	Pb, Ni, Cd	25	100	7	853.89	196.87, 90.90, 163.21	Langmuir, Freundlich, Redlich-Peterson, Sips	–	ΔH, ΔS, ΔG	^30^

In the case of waste polycarbonate, the purity of these plastics is very critical. Recycling plastics via mechanical methods has economic and environmental advantages. For this reason, various methods, such as flotation, are applied. Flotation is recognized as the promising procedure for separating plastics with a size of 2–4 mm and densities of more than 1 g cm^−3^ owing to cost-effectiveness and simple procedures. It is based on the interaction between the air bubbles and the polymer surface^20,21^.

According to the advantages and chemical stability results of polymeric adsorbents, the purpose of this research is to use waste polycarbonate for helping to the environment for two main reasons including (1) reducing the waste components containing polycarbonate, and (2) removing the hazardous heavy metal ions from the water sources. The waste polycarbonate was hypercrosslinked to produce the polymeric adsorbent. Isotherm equations like Langmuir, Freundlich, Redlich–Peterson, and Temkin are applied to detect the adsorbent behavior. In addition, the kinetic models are applied to study how the adsorption rate is changed. The temperature, pH, and reusability of the adsorbent are examined. Finally, the thermodynamic variables are calculated to find the spontaneous or nonspontaneous and exothermic or endothermic nature of the lead and cadmium ions. The novelty of this work is collecting the waste polycarbonates from the environment for two main reasons. First, preventing the pollution of soil and water sources with the waste polycarbonate, which can generate the serious disease for humans. Second, converting these waste materials into adsorbents for depleting the perilous metallic ions. The advantage of this work is utilizing the waste polycarbonate as the precursor instead of buying the high-purity polymers, which have a higher price than the waste polycarbonate, which can be feasible for application at the industry centers. The disadvantage of this work is its lower uptake capacity, which returns to the purity of these waste polycarbonates, and this case can force us to graft some effective materials to this waste polycarbonate to improve their ability to eliminate the hazardous heavy metal ions. In addition, the lower purity of waste polycarbonate than the raw polycarbonate possibly generates some challenges in the Friedel–Crafts reactions for the cross-linking process.

The novelty of this work is categorized into three parts which are listed following: (1) Using waste materials with the base of polycarbonate as the precursors for cross-linking their networks, (2) Calculating the mass transfer parameters for querying more the adsorption behaviors, (3) Employing the complicated multi-component isotherm equations for inspecting the adsorption of lead and cadmium ions, and (4) Investigating the reusability of the hyper-cross-linked waste polycarbonate.

## Empirical

### Necessary components

The essential polycarbonate has been collected from the compressed disk (CD) pieces that have utilized as the polycarbonate of the HCP. The considered solvent and crosslinking agents were dichloromethane (DCM) and Formaldehyde Dimethyl Acetal (FDA), respectively. Iron chloride catalyzed the Friedel–Crafts reaction. Additionally, the entire applied components have been collected from Merck. Acid and base have been exploited for tuning the pH of media. Cd (NO_3_)_2_ and Pb(NO_3_)_2_ were exploited to prepare the metal contaminant solution. The details of the necessary components have presented in Table 2.

**Table 2:** Details of the chemical components in the experiments.

Component	IUPAC name	Chemical formula	Molecular weight (g mol^−1^)	CAS number
Polycarbonates	Poly(Bisphenol A Carbonate)	C_16_H_18_O_5_	272.29	25037-45-0
FDA	Methylal	C_3_H_8_O_2_	76.11	109-87-5
DCE	dichloromethane	CH_2_Cl_2_	84.93	97002-70-5
Iron(III) chloride	Iron trichloride	FeCl_3_	162.20	7705-08-0
Lead(II) nitrate	Lead(II) nitrate	Pb(NO_3_)_2_	331.2	7722-76-1
Cadmium(II) chloride	Cadmium dichloride	CdCl_2_	183.32	10108-64-2

### Synthesis of hypercrosslinked waste polycarbonate

In the primary stages of synthesis, 30 ml of solvent has been added into the flask, and 0.01 mol of monomer and 0.03 mol of cross-linking agent have been introduced. The components were combined at room temperature. Then, 0.03 mol of catalyst was incorporated into the blend and agitated for 3 h at 45 °C. Afterward, the temperature has been promoted to 80 °C, and exposed to the reflux reaction for 11 h. For finishing the Friedel–Crafts process, the mixture has been blended and continuously rinsed with the ethanol until the remained solution became colorless. The Soxhlet extraction has been exploited for removing the unreacted components from the sorbent for 12 h at 75 °C. For making rigid obtained sorbent, the constructed resin stayed inside the vacuum oven for 8 h at 80 °C, and the brown powder was observed^17,31^.

### Metal ions adsorption

The adsorption test was examined in a batch system. Initially, a certain amount of resin (0.03 g) was loaded inside the Erlenmeyer containing 100 ml of different metallic concentrations (20, 40, 60, 80, and 100 mg/L) in the diverse heat degrees (20, 35, 50, 65, and 80 °C) and pH contents (6, 7, 8, 9, and 10). In order to adjust the pH of ambient, 0.01 mol/L HCl and 0.05 mol/L NaOH have been used. The head of the Erlenmeyer was absolutely covered to impede the volatilities. The solution was mixed at the rate of 140 r/min for 24 h. The final concentration of metallic ions was determined with ICP-OES (Inductively Coupled Plasma Optical Emission spectroscopy). Also, it is essential to point out that the radius or diameter of the pores was calculated N_2_ adsorption–desorption at 77 K using a porosity analyzer (Micromeritics, ASAP2020, USA). Prior to the analyses, the adsorbents were subjected to nitrogen at 383 K for 12 h under a vacuum.

**Figure 1:**
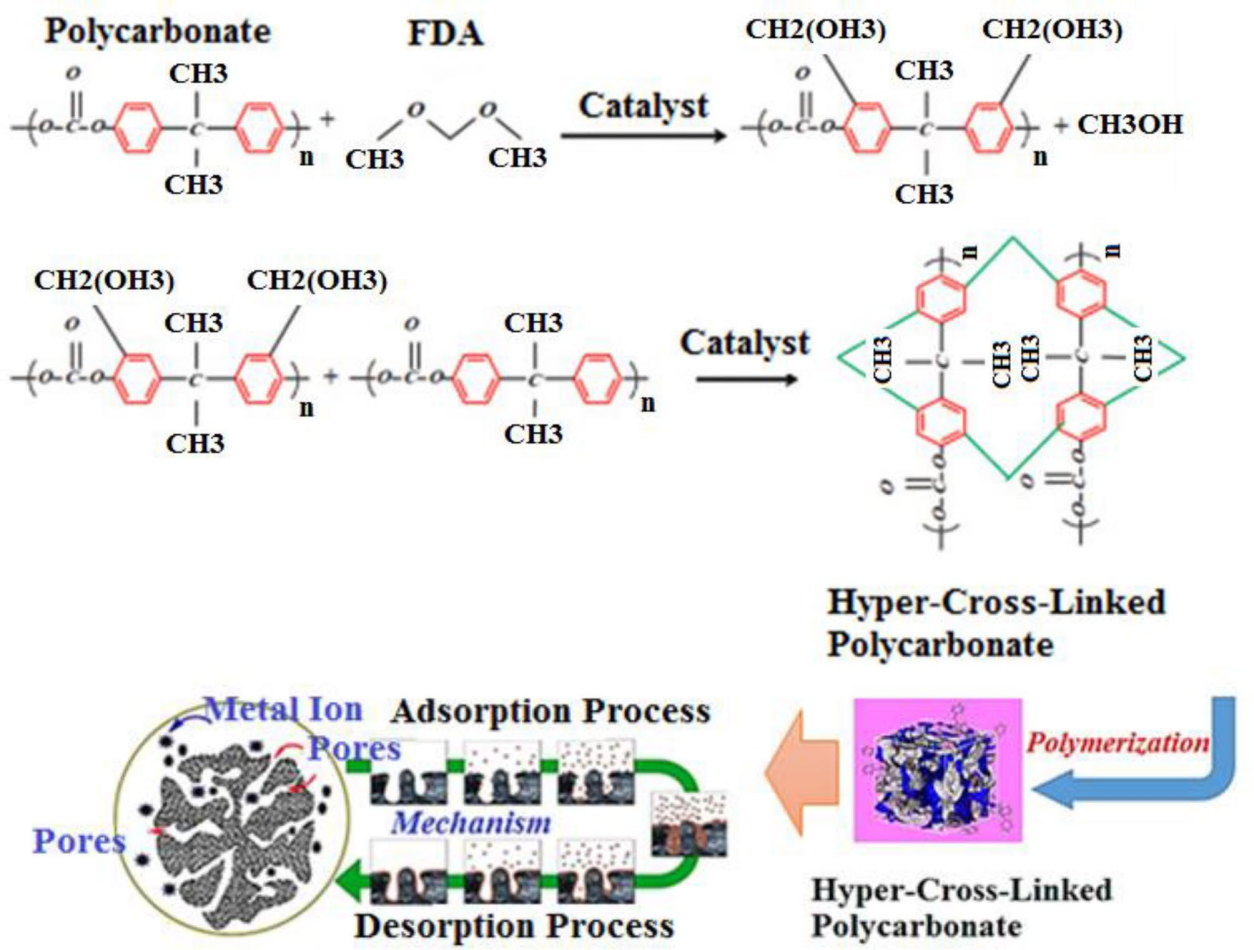
The chemical reaction of waste polycarbonate with the solvents and catalyst in the Friedel–Crafts synthesis method and adsorption process^32^.

The mechanism of synthesis and adsorption is illustrated in Fig. 1. Besides, Fig. 1 displays that the adsorption of metal ions mainly occurs inside the pores.

Measuring the adsorption amount has more significance for identifying the tendency of sorbate to diffuse into the cavities of the adsorbent. q_e_ has calculated employing the Eq. (1)^29,33,34^:

1 $$q_{e} = \frac{{\left( {C_{o} - C_{e} } \right) V}}{m}$$

C_o_ is the initial metallic ion concentration, C_e_ is the metallic ion concentration at equilibrium (mg L^−1^), m is the mass of the resin (g) and V is the volume (L) of the solution. The average relative error (ARE) and coefficient of determination (R^2^) have been determined with respect to Eq. (2) and Eq. (3), respectively. The mass transfer coefficient (K_L_) and mass flux (N) are measured with respect to Eq. (4) and Eq. (5), respectively. Also, C* refers to saturated concentration:

2 $$\% \,ARE = \left[ {\sum\limits_{i = 1}^{N} {\left| { \, \left( {\left( {q_{{}}^{\exp } - q_{{}}^{cal} } \right) \, /q_{{}}^{\exp } } \right) \, } \right|/N} } \right] \times 100$$

3 $$R^{2} = \frac{{(q_{\exp } - \overline{q}_{calc} )^{2} }}{{\sum\nolimits_{i = 1}^{n} {((q_{\exp } - \overline{q}_{calc} )^{2} + (q_{\exp } - q_{calc} )^{2} )} }}$$

4 $$K_{L} = \frac{{N_{{}} }}{{\left( {C_{o} - C_{e} } \right)}}$$

5 $$N = \frac{1}{3}\left( {C_{o} - C_{{}}^{*} } \right)\left( {1 - \exp \frac{{ - Dn^{2} \pi^{2} t}}{{r_{S}^{2} }}} \right)$$

The diffusion coefficient (D) is calculated with respect to Eq. (6) to Eq. (10). In the following equations, θ is the time, r_S_ is the average diameter of the pores of the sorbent, and q_t_ is the uptake capacity of the sorbent at time t.

6 $${\text{F}} = 1 - \frac{6}{{\uppi ^{2} }}\sum\limits_{{{\text{n}} = 1}}^{\infty } {\frac{1}{{{\text{n}}^{2} }}} \exp \left( { - \frac{{{\text{Dn}}^{2}\uppi ^{2}\uptheta }}{{{\text{r}}_{{\text{S}}}^{{2}} }}} \right)$$

7 $${\text{F}} = 1 - \frac{6}{{\uppi ^{2} }}\sum\limits_{{{\text{n}} = 1}}^{\infty } {\frac{1}{{{\text{n}}^{2} }}} \exp \left( { - {\text{Bn}}^{2}\uptheta } \right)$$

8 $${\text{F}} = \frac{{{\text{q}}_{{\text{t}}} }}{{{\text{q}}_{{\text{e}}} }}$$

9 $${\text{B}} = \frac{{{\text{D}}\uppi ^{2} }}{{{\text{r}}_{{\text{S}}}^{{2}} }}$$

10 $${\text{B}}\uptheta = 0.4977 - \ln (1 - {\text{F}})$$

## Result and discussion

### Adsorbent characterization

**Figure 2:**
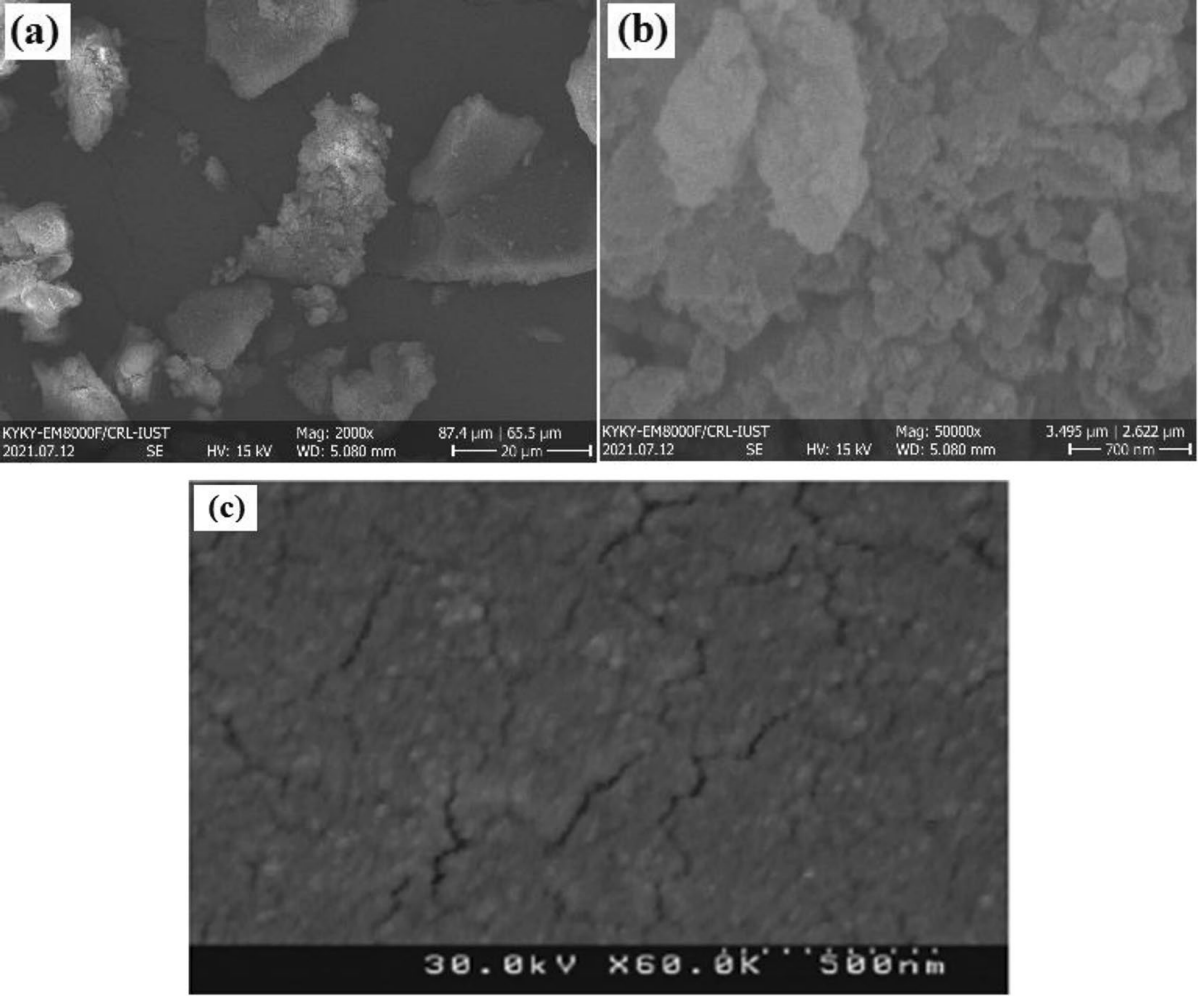
The FE-SEM images of hypercrosslinked waste polycarbonate with the magnification of (**a**) 20 μm and (**b**) 700 nm. (**c**) The FESEM image of hypercrosslinked waste polycarbonate after the adsorption.

The structure features of hypercrosslinked waste polycarbonate were investigated using FE-SEM, FTIR, EDS, TGA, and BET analyses. Figure 2a,b represent the FE-SEM micrographs of the as-prepared hypercrosslinked waste polycarbonate nanoparticles. It has resulted that the sorbent contains spherical and coarse units and the mean diameter of the fabricated nanoparticles was almost 7.85 nm. It was clear that the synthesized adsorbent has an asymmetric architecture. The pores of the hypercrosslinked polycarbonate were irregular. High porosity has been observed in the hypercrosslinked polycarbonate. With respect to Fig. 2b, it was observed that the pores stuck together. Therefore, there were adequate empty places for the collection of metal ions. According to Fig. 2c, it was observed that the hypercrosslinked waste polycarbonate plane was flat after the sorption and demonstrated that the Cd(II) and Pb(II) ions surrounded the holes.

**Figure 3:**
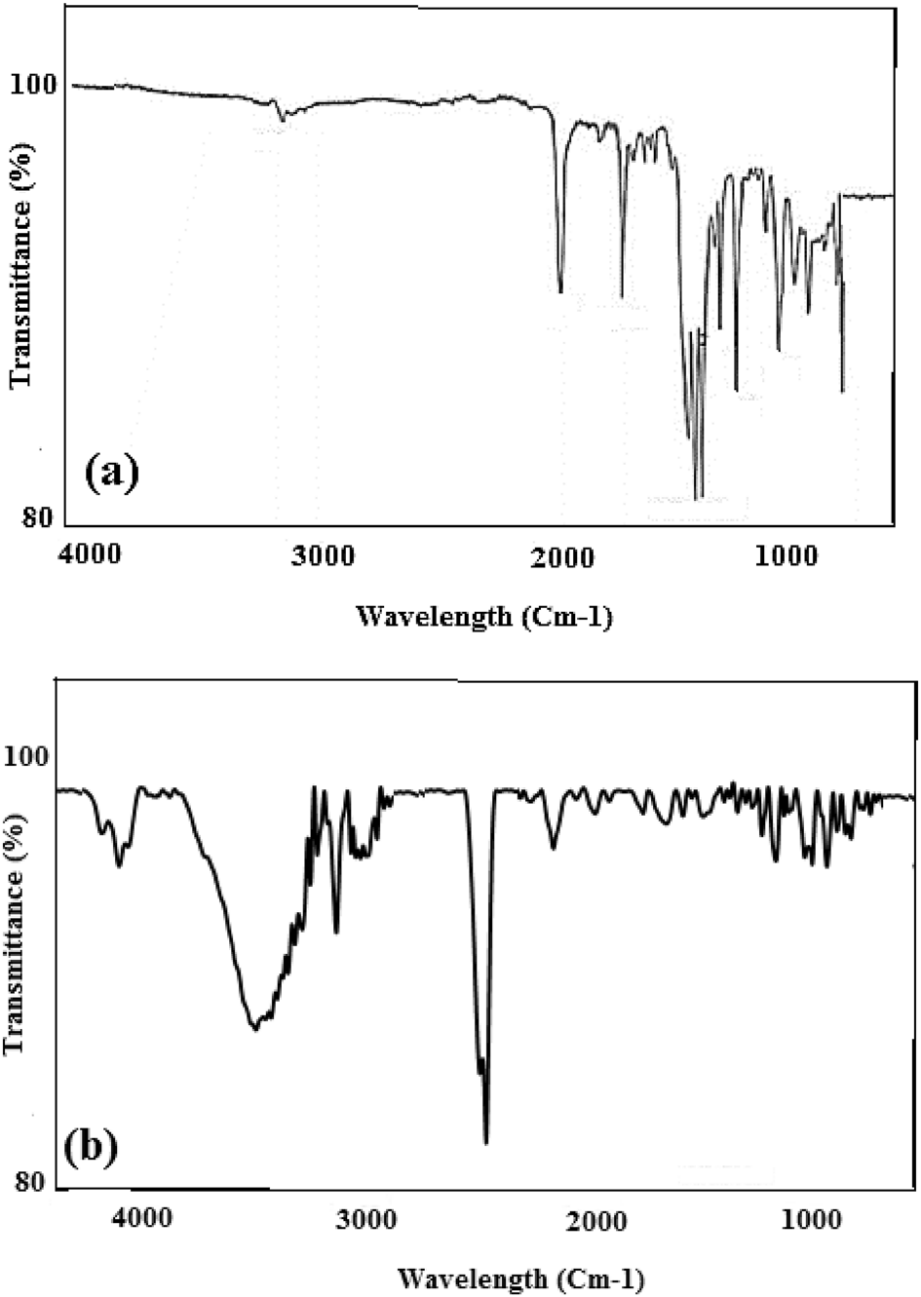
The FTIR pattern for the (**a**) polycarbonate, and (**b**) hypercrosslinked waste polycarbonate.

The FTIR test for the polycarbonate was disclosed in Fig. 3a. The peaks of polycarbonate have been found at the wavelength of 2966, 1770, 1504, 1250–1100, 1080, and 1014 cm^−1^ that corresponds to the stretching vibration of C–H bonds of CH_3_ group, carbonate group (C = O) vibration, ring C–C vibration from the two phenol ring, stretching deformations of asymmetric O–C–O carbonate group, CH_3_ vibration, and symmetric O–C–O carbonate group, respectively. Compared with Fig. 3b, the peaks of carbonate groups (such as 1014, 1250–1100, and 1770 cm^−1^) have disappeared because the carbonate group was involved in the cross-linking reaction. Figure 3b depicts the FTIR test of the hypercrosslinked waste polycarbonate. The FTIR spectra of the adsorbent displayed that the vast intensity revealed at 3200–3500 cm^−1^ was due to the O–H vibration. The intensity at 1658 cm^−1^ was owing to the C–Cl stretching. The C = C stretching in the benzene rings was observed at 1658 cm^−1^. The slight intensity at 698–759 cm^−1^, could be ascribed to the bending of the C–H in the aromatic ring. The intensity at 2936 cm^−1^ was owing to –CH_2_– groups in the Friedel–Crafts procedure. Besides, the peak at 3752 cm^−1^ was ascribed to the aliphatic C–H group’s presence.

**Figure 4:**
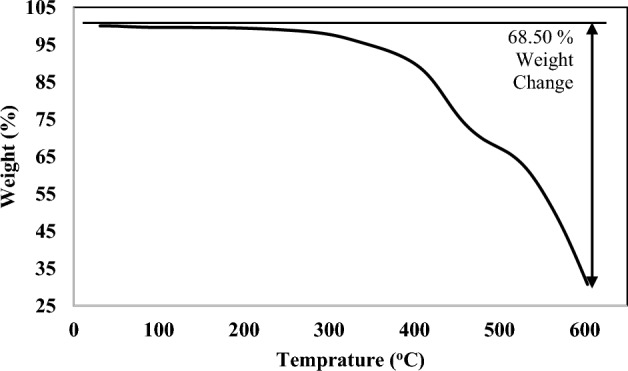
The TGA diagram of the hypercrosslinked waste polycarbonate.

The TGA diagram of the adsorbent is presented in Fig. 4. It showed that the content of the resin declined mildly at 300 °C (5%), which could be due to the release of solvent and water. The sorbent started to decompose at 450 °C, disclosing its good thermal stability because of the sticking of the carbon atoms. Moreover, it could be derived that the 600 °C was not an appropriate heat degree since the large amount of the resin deteriorated (68.50%).

**Figure 5:**
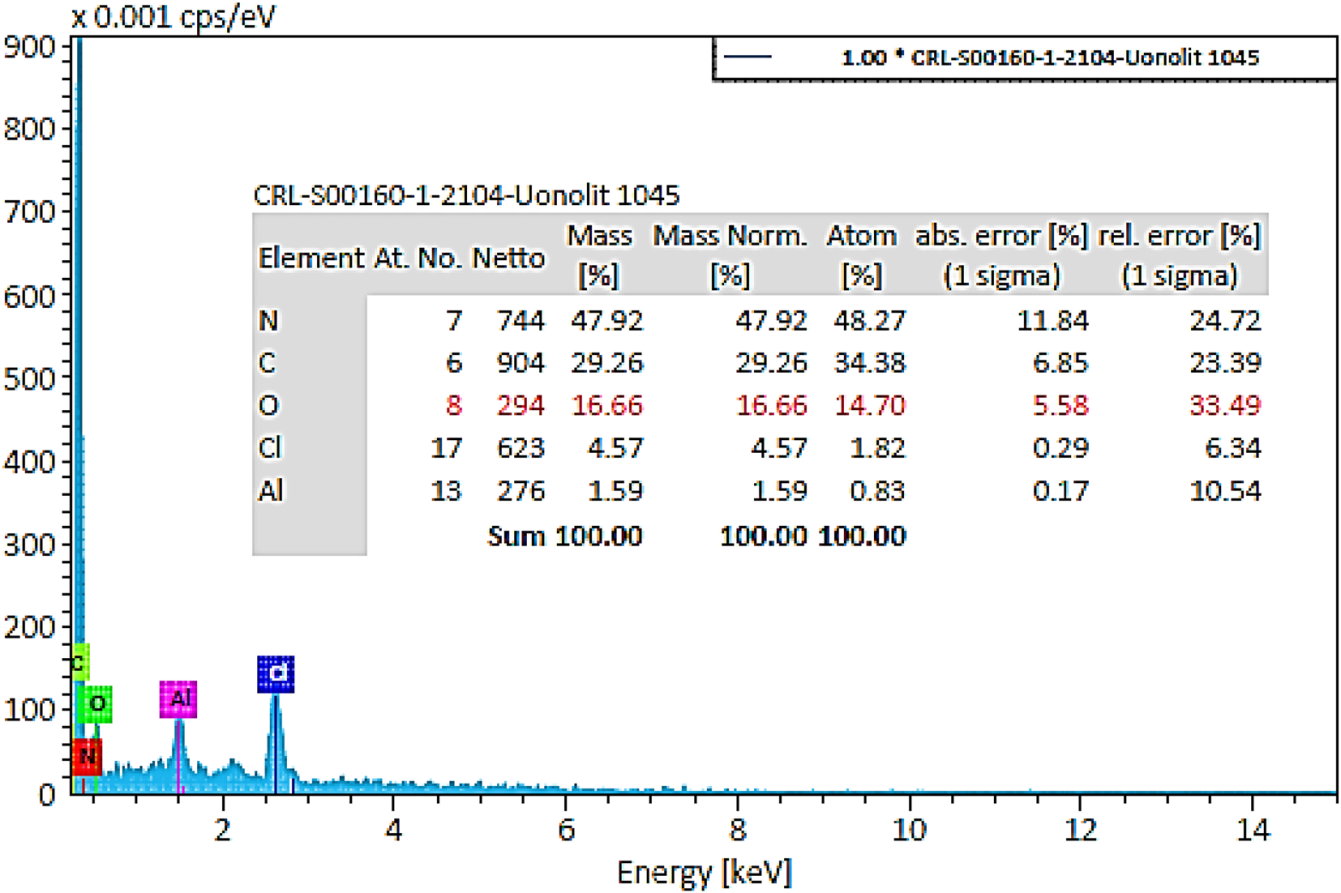
The EDS results of hypercrosslinked waste polycarbonate.

According to Fig. 5, the compositions of waste polycarbonate contained oxygen (49.28%), carbon (42.63%), and aluminum (8.09%). The presence of oxygen and carbon was ascribed to the Friedel–Crafts synthesis method. Additionally, aluminum was the main composition in the CD wastes, hence it was detected in the EDS test.

**Figure 6:**
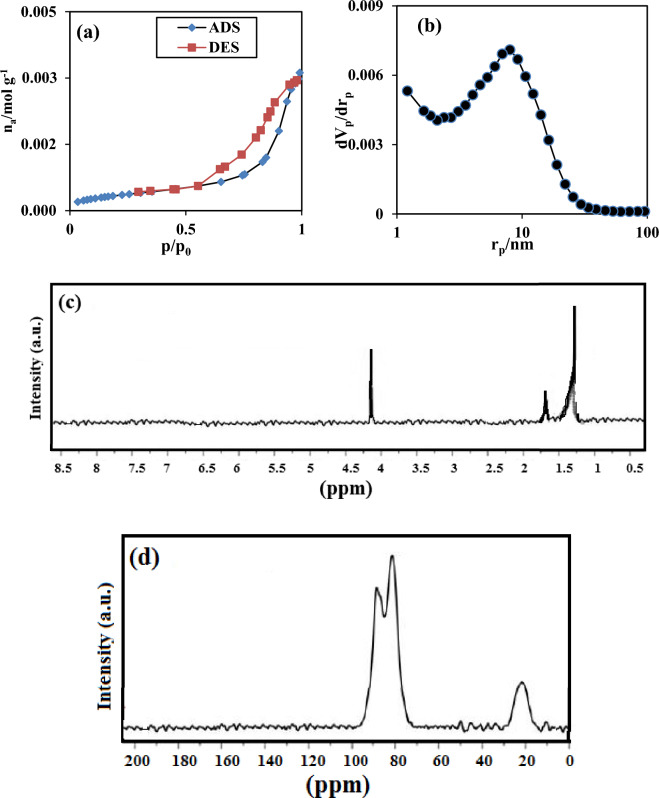
The diagram of (**a**) nitrogen adsorption–desorption, (**b**) pore size distribution of the hypercrosslinked waste polycarbonate, NMR spectrum of (**c**) Polycarbonate, and (**d**) Hypercrosslinked Polycarbonate.

The N_2_ adsorption–desorption in Fig. 6a depicted the collection of a large number of macro-pores on the plane of the sorbent at a wide pressure proportion (P/P_o_ = 0.93). Moreover, a wide peak was detected at 7.85 nm in Fig. 6b disclosing the nanoscale network of the adsorbent since the diameter of the holes was lesser than 50 nm. As observed in Fig. 6b, the intensity of the peak was strong at the radius of 7.85 nm which implied that most of the pores had this diameter. The BET of the resin was listed in Table 3, and the surface area of the resin has been determined at 813.810 m^2^/g. Table 3 revealed that the average hole width was vast enough (15.720 nm) for receiving the cadmium and lead. The 1H-NMR spectrum for the polycarbonate and hypercrosslinked polycarbonate were illustrated in Fig. 6c,d, respectively. In the 1H-NMR spectrum of polycarbonate, there were three peaks at 4.20, 1.62, and 1.25 ppm, respectively. The peaks at 4.20, 1.62 and 1.25 ppm corresponded to C–O, C–C, and C = O bonds in the chain of polycarbonate, respectively. After the Friedel–Crafts process, the benzene rings of polycarbonate were knitted together which influenced the 1H-NMR spectrum of the hypercrosslinked polycarbonate. Likewise, three peaks were observed in the 1H-NMR spectrum of the hypercrosslinked polycarbonate at 134, 123, and 33 ppm which belongs to the C = O, aromatic carbon of benzene ring, and methylene carbon that was formed via the Friedel–Crafts reaction.

**Table 3:** The single and multi-component isotherm models.

Model name	Single-component isotherm	Multi-component isotherm
Langmuir	${\text{q}}_{{\text{e}}} = \frac{{{\text{q}}_{{\text{m}}} {\text{K}}_{{\text{L}}} {\text{C}}_{{\text{e}}} }}{{1 + {\text{K}}_{{\text{L}}} {\text{C}}_{{\text{e}}} }}$	${\text{q}}_{{\text{e,i}}} = \frac{{{\text{q}}_{{\text{m,i}}} {\text{K}}_{{\text{i}}} {\text{C}}_{i} }}{{1 + \sum\limits_{{{\text{j}} = 1}}^{{\text{N}}} {{\text{K}}_{{\text{j}}} {\text{C}}_{{\text{j}}} } }}$
Freundlich	${\text{q}}_{{\text{ e}}} = {\text{K}}_{{\text{F}}} \,{\text{C}}_{{\text{e}}}^{{\text{ m}}}$	${\text{q}}_{{\text{e,i}}} = {\text{K}}_{{\text{F,i}}} {\text{C}}_{{\text{i}}} \left( {\sum\limits_{{{\text{j}} = 1}}^{{\text{N}}} {{\text{a}}_{{{\text{ij}}}} {\text{C}}_{{\text{j}}} } } \right)^{{{\text{m}}_{{\text{i}}} - 1}}$
Redlich–Peterson	${\text{q}}_{{\text{e}}} = \frac{{{\text{a}}\,{\text{C}}_{{\text{e}}} }}{{1 + {\text{bC}}_{{\text{e}}}^{\upbeta } }}$	${\text{q}}_{{\text{e,i}}} = \frac{{{\text{a}}_{{\text{i}}} {\text{C}}_{{\text{i}}} }}{{1 + \sum\limits_{{{\text{j}} = 1}}^{{\text{N}}} {{\text{b}}_{{\text{j}}} {\text{C}}_{{\text{j}}}^{{\upbeta _{{\text{j}}} }} } }}$

### Isotherm and kinetic modeling

**Figure 7:**
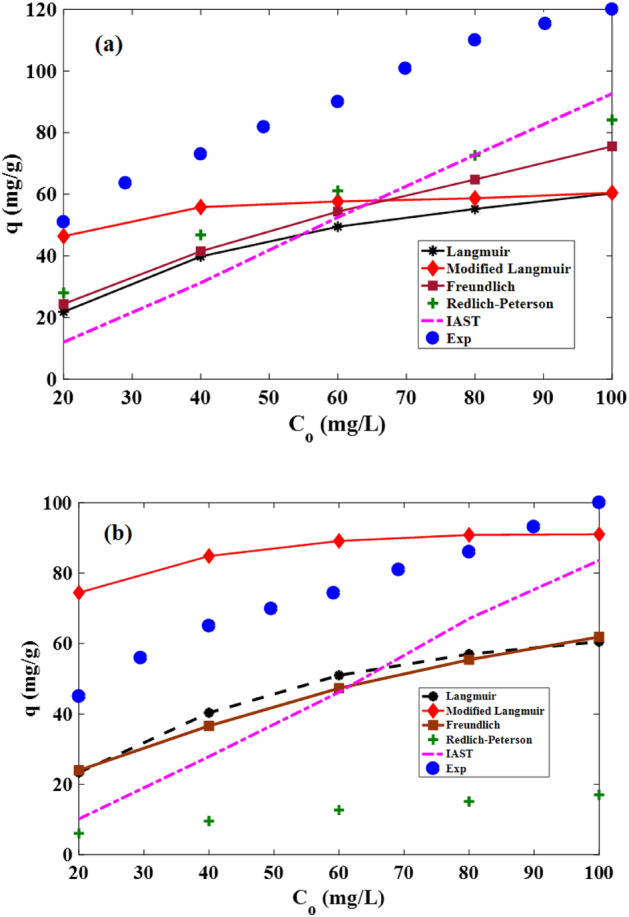
The diagram of empirical and predicted results using multi-component isotherm relations at normal temperature for (**a**) lead and (**b**) cadmium ions.

**Figure 8:**
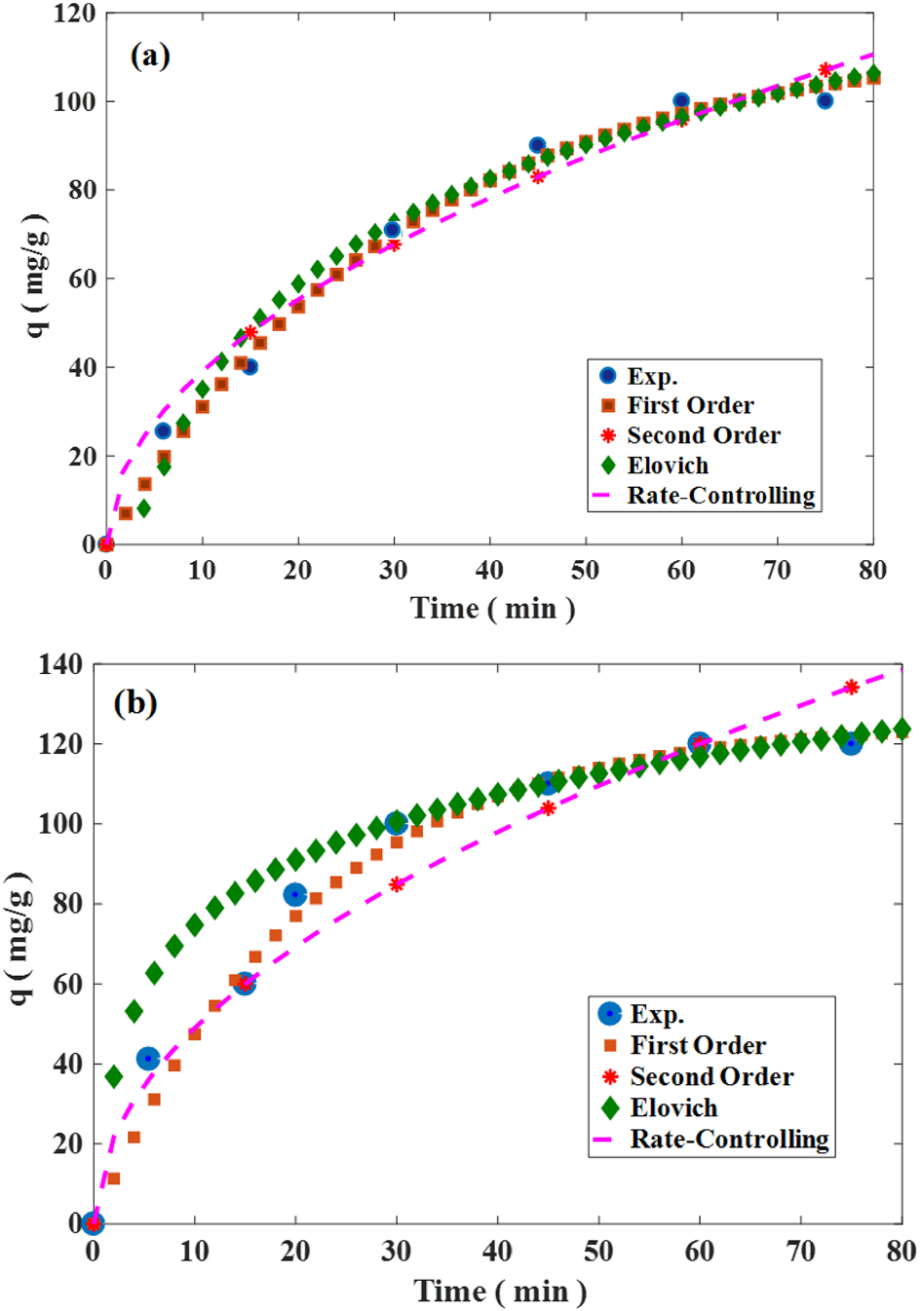
Experimental and kinetic models results for (**a**) Cd and (**b**) Pb ions.

Isotherm expressions create relations between the sorbent and the solute at a certain temperature^35^. For calculating the uptake of metallic ions in the binary solutions, the isotherm parameters in the single-component system were determined in Table 4. According to Table 4, Freundlich was the most appropriate model. The Freundlich model showed that the multiple heterogeneous layers belong to the resin for Cd(II) adsorption. The required isotherm relations for the binary solutions are presented in Table 4. According to Fig. 7a,b, the modified-Langmuir and IAST-Freundlich had suitable adaption with the empirical results for lead and cadmium, respectively. With respect to Table 4, the quantity of n referred to as the Freundlich constant. It was greater than unity at the room heat degree which implied the adsorption procedure was conducted promisingly. Figure 8 referred to the saturation moment of these two metal ions which was 60 min. The terminal concentration of Cd and Pb was not altered after this moment. The uptake capability at the equilibrium moment of Cd and Pb was found at 100 and 120 mg/g, respectively. In addition, kinetic equations were utilized including pseudo-first-order, pseudo-second-order, Elovich, and rate-controlling. The parameters of kinetic relations were written in Table 5. After comparing, pseudo-second-order could fit with the experimental results implying the physical interaction has occurred between adsorbent and metallic ions. Also, the highest value of R^2^ in the Elovich proved the heterogeneous surface of the adsorbent. Moreover, the slight value of the equilibrium moment of these metallic ions disclosed the economic status of the present constructed sorbent^36^.

**Table 4:** The parameters of isotherm models for hypercrosslinked waste polycarbonate at 20 °C.

Metal ion	Model	Parameter	Unit	Value	R^2^	ARE
Single-system parameters						
Cd	Langmuir	q_m_	mg/g	140.326	0.981	0.046
		K_L_	L/mg	0.021		
	Freundlich	n	–	2.060	0.995	0.025
		K_F_	mg^(1−m)^ . L^m^/g	10.446		
	Redlich–Peterson	α	L/mg	3.2e6	0.992	0.025
		β	–	0.515		
		K_R_	(L/mg)^β^	3.3e7		
Pb	Langmuir	q_m_	mg/g	193.254	0.992	0.038
		K_L_	L/mg	0.016		
	Freundlich	n	–	1.836	0.998	0.014
		K_F_	mg^(1−m)^ . L^m^/g	9.862		
	Redlich–Peterson	α	L/mg	1.7e7	0.998	0.014
		β	–	0.455		
		K_R_	(L/mg)^β^	1.7e8		
Multi-component system						
Metal ion	Model	Parameter	Unit	Value	R^2^	ARE
Cd	Langmuir	Same as single-system	Same as single-system	Same as single-system	0.990	0.0015
	Freundlich	a_Cd–Pb_	–	0.980	0988	0.0033
		a_Cd–Cd_		1		
	Redlich–Peterson	Same as single-system	Same as single-system	Same as single-system	0.951	0.051
Pb	Langmuir	Same as single-system	Same as single-system	Same as single-system	0.975	0.0051
	Freundlich	a_Pb–Cd_	–	1.02	0.995	0.0023
		a_Pb–Pb_		1		
	Redlich–Peterson	Same as single-system	Same as single-system	Same as single-system	0.932	0.032

**Table 5:** Kinetic parameters for sorption of the metal ions using hypercrosslinked waste polycarbonate.

Metal	Model	Parameter	Unit	Value	R^2^	ARE%
Cd	Pseudo-First-Order	q_1_	mg g^−1^	114.291	0.995	0.049
		K_1_	min^−1^	0.032		
	Pseudo-Second-Order	q_2_	mg g^−1^	160.183	0.995	0.036
		K_2_	g mg^−1^ min^−1^	1.59e − 40		
	Elovich	α_E_	mg g^−1^ min	0.008	0.997	0.038
		β_E_	mg g^−1^	34.281		
	Rate-controlling	k_id_	mg g^−1^ min^−1/2^	12.367	0.988	0.084
Pb	Pseudo-First-Order	q_1_	mg g^−1^	125.815	0.995	0.046
		K_1_	min^−1^	0.047		
	Pseudo-Second-Order	q_2_	mg g^−1^	160.535	0.998	0.030
		K_2_	g mg^−1^ min^−1^	2.89e − 4		
	Elovich	α_E_	mg g^−1^ min	0.0099	0.968	0.053
		β_E_	mg g^−1^	37.961		
	Rate-controlling	k_id_	mg g^−1^ min^−1/2^	15.497	0.980	0.065

### Thermodynamic modeling

**Figure 9:**
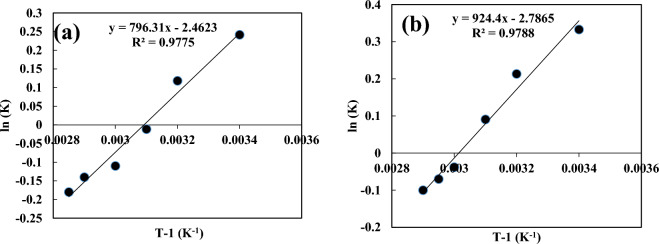
ln (K) vs. T^−1^ for (**a**) cadmium, (**b**) lead ions.

The energy exchange of the resins is described by the thermodynamic constants. Equation (11) was Van Hoff’s equation which was used for calculating enthalpy (ΔH°), and entropy differentials (ΔS°). Subsequently, Eq. (12) was applied to determine the Gibbs free energy differentials (ΔG°). The negative sign of ΔS° of these metallic ions referred to the heat released in the adsorption process. Additionally, the lower quantity of ΔS° from 40 kJ/mol implied the sorption process was physical. The negative sign of Gibbs free energy differentials of Cd and Pb at 20, 40, and 60 °C exhibited the spontaneous mode of adsorption, and the quantity of ΔG° enhanced by increasing the heat degree, which explained the heat degree promoting, is not appropriate. ΔG° was positive at 80 °C for metallic ions revealing the sorption was not spontaneous. The ln (K) with respect to T^−1^ is shown in Fig. 9. The value of K was determined via plotting ln q_e_/C_e_ with respect to q_e_. The quantity of Gibbs free energy changes for the removal of lead ions was higher than cadmium ions. Also, the evaluations displayed that the sorption of lead ions onto the sorbent needs more energy, and the process was performed more spontaneously relative to the cadmium ions^37^. The thermodynamic parameters are listed in Table 6.

11 $$\ln \,K_{d} = \frac{{\Delta S^{0} }}{R} - \frac{{\Delta H^{0} }}{RT}$$

12 $$\Delta G^{0} = \Delta H^{0} - T\Delta S^{0}$$

**Table 6:** Thermodynamic constants of heavy metal ions adsorption by hypercrosslinked waste polycarbonate.

Metal ion	ΔH (kJ/mol)	ΔS (kJ/mol.K)	ΔG (kJ/mol)	T (°C)
Cd(II)	− 6.62	− 0.0204	− 0.6397	20
			− 0.3337	35
			− 0.0277	50
			0.2783	65
			0.6455	80
Pb(II)	− 7.68	− 0.0231	− 0.9082	20
			− 0.5617	35
			− 0.2152	50
			0.1313	65
			0.5471	80

### Effect of metal content, temperature and pH

**Figure 10:**
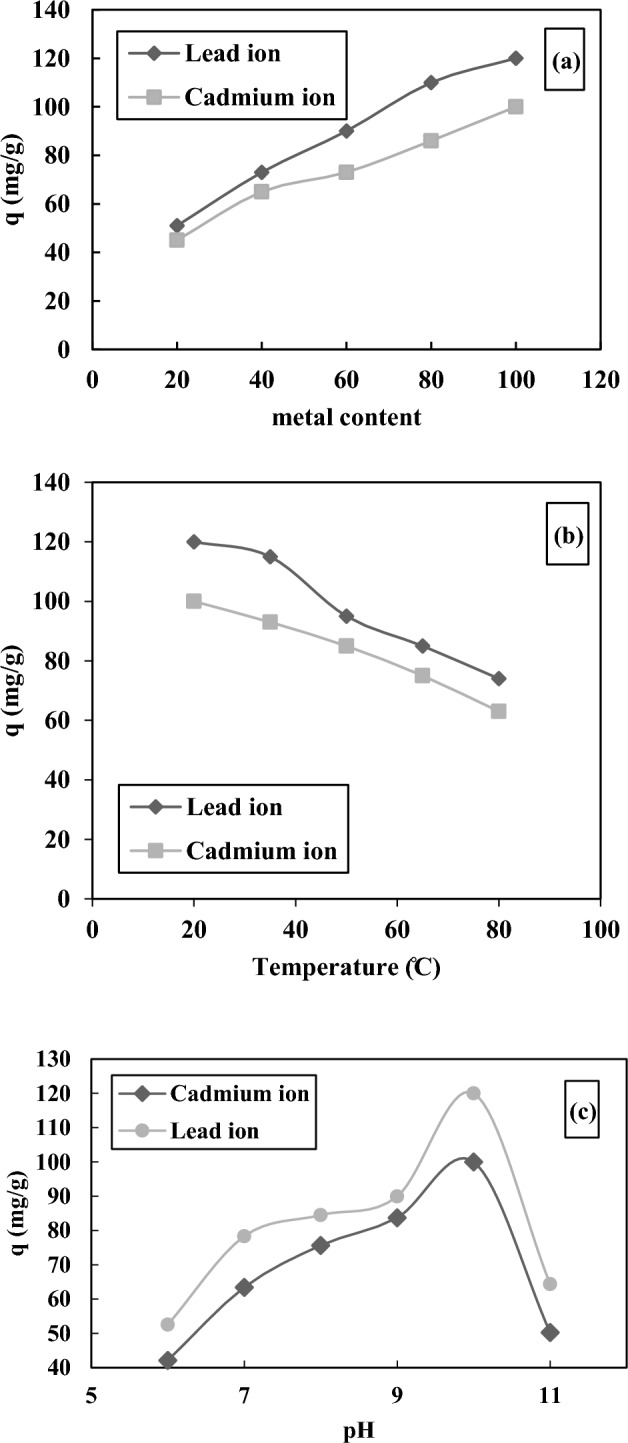
The diagram for the study the effect of the (**a**) metal content, (**b**) temperature, and (**c**) pH.

Figure 10a implied that the initial concentration enhancement had a positive impact on the uptake capacity, in which the uptake capacity of lead ions was enhanced from 51 to 120 mg/g by increasing the metal content from 20 to 100 mg/L, respectively. Also, the uptake capacity of cadmium ion was enhanced from 45 to 100 mg/g by increasing the metal content from 20 to 100 mg/L, respectively. This case was attributed to the mobility of more metallic ions toward the vacant cavities. Figure 10b revealed the influence of temperature on the adsorption capability. Regarding this figure, the uptake capacity was dropped with the enhancement of temperature, which could be due to the damaging structure of the adsorbent during the temperature increase. pH is an important variable in the sorption of metallic particles. Figure 10c depicts the influence of pH on the metallic particles' adsorption. The pH contents were tuned 6, 7, 8, 9, 10, and 11 utilizing 0.05 M HCl and 0.05 M NaOH. Regarding Fig. 10c, the sorption capability is enhanced by elevating the solution pH. As pH enhanced, the electrostatic interactions among the metal ions and HCP increased because the H_3_O^+^ contents decreased, thus, the metal ions could occupy the cavities of resin more easily. The highest value of adsorption capability for these ions has been found at pH 10. At pH > 10, the uptake declined due to the creation of metal hydroxides.

### Mass transfer results

Table (7) explains that the value of D (Diffusion Coefficient) for Pb was greater than Cd. In order to justify the previous sentence, this explanation could be used: "*The harder and denser the metal, the smaller the diffusion coefficient*". By comparing the boiling point, it was concluded that the boiling point of Pb (1749 °C) was greater than Cd (767 °C). Thus, Pb was more robust than Cd implying the value of D for Cd was bigger^38^. Additionally, the mass transfer coefficient (K_L_) of Pb was greater than Cd because of the greater uptake capability of Pb relative to Cd (Table 7), or in other words, the rate of Pb for filling the channels was greater than Cd. According to Table 7, the flux (N) and mass transfer coefficient increased with time, because with respect to the mass transfer flux relation, which was the proportion of migrated particles to the unit of moment and area, it could be said that by promoting the moment, higher amount of channels of resin were filled with ions, which caused to a decline in the contact area of the adsorbent and also the mass transfer of metallic ions.

**Table 7:** The mass transfer parameters in the two-component system for cadmium and lead ions.

T (min)	q_t_ (mg/g)	C_Ae_ (mg/L)	F	B	D × 10^20^ (m^2^/s)	N × 10^21^ (mol/m^2^ s)	K_L_ × 10^15^ (m/s)
Lead ions							
15	60	88	0.5000	0.0013	0.1504	0.1507	0.1507
30	100	80	0.8333	0.0013	0.1392	0.2512	0.1722
45	110	78	0.9167	0.0011	0.1269	0.2763	0.2028
Cadmium ions							
15	40	92	0.4000	0.0011	0.1776	0.0545	0.0831
30	70	86	0.7000	0.0009	0.1708	0.0954	0.0867
45	90	82	0.9000	0.0010	0.1463	0.1226	0.1054

### Multicomponent adsorption mechanism

**Figure 11:**
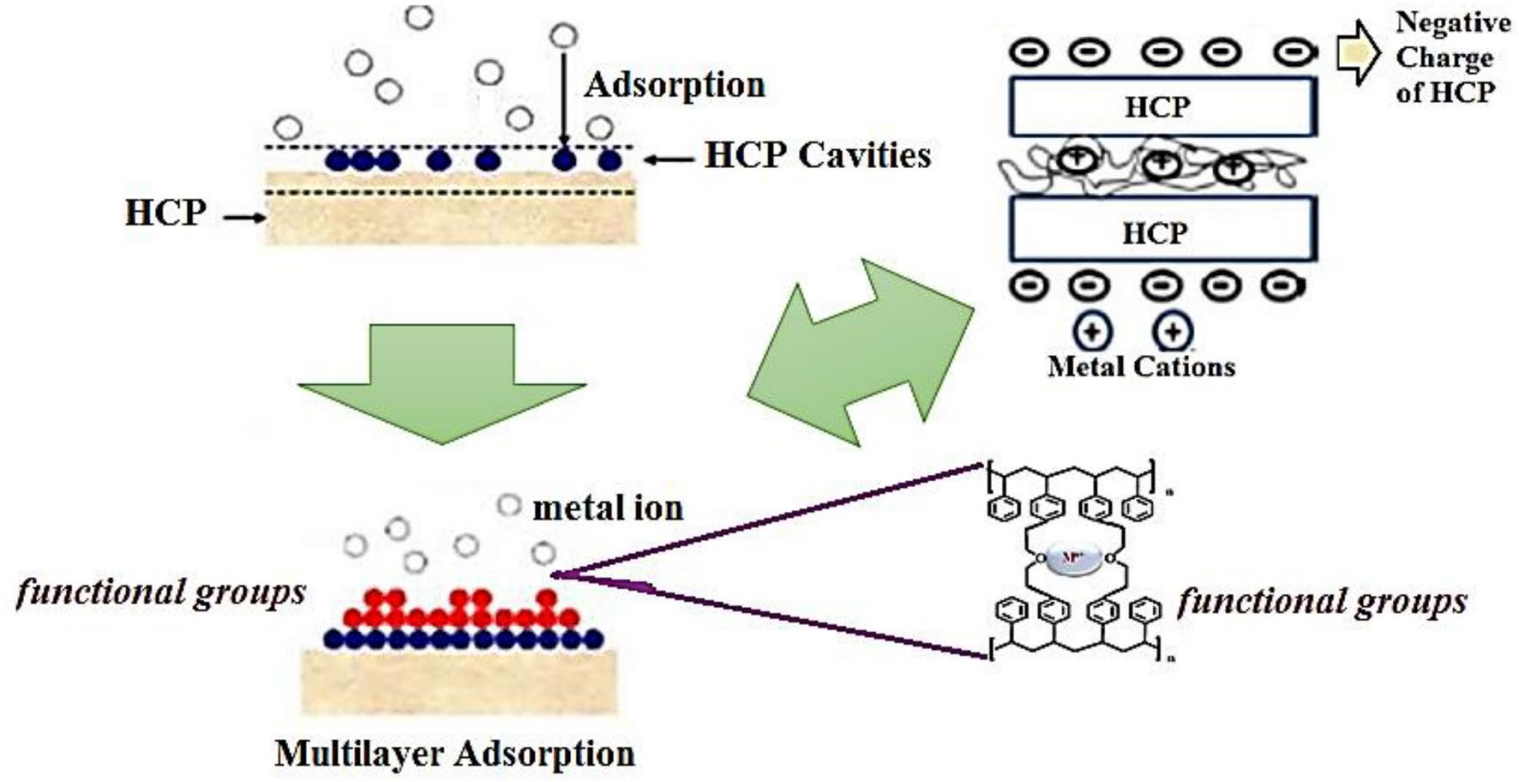
The schematic of adsorption mechanism on porous hypercrosslinked adsorbent.

The main principles in the sorption mechanism were (1) Chelation between functional moieties and sorbent; (2) Presence of ‘cation-π’ among the aromatic groups and metallic particles; and (3) Presence of several holes and supreme surface area of the resin^39^. According to the FTIR image, the C–O–C and C–H in the aromatic groups had a crucial aspect in the bond formation among metallic ions and resin. In this case, cadmium and lead ions are generated and joined with the oxygen of the C–O–C moiety. Moreover, the anionic part of the metallic salt binds with the hydrogen of the C–H in the aromatic group. The adsorption was conducted by releasing heat that could be owing to the bond production in the interaction of metallic ions and HCP because the enthalpy changes in cadmium (− 6.62 kJ/mol) and lead ions (− 7.68 kJ/mol) are negative. The chelation behavior of bond generation was carried out spontaneously because of the negative sign of Gibbs free energy changes at the temperature of 20, 35, and 50 °C for lead and cadmium ions, and privately at a slight temperature^40^. The adsorption selectivity was the comparative disposition of metallic ions towards the metallic solution and resin which was due to the inherent property and content of the functional moieties, and a differential in the size of the metallic ions, which explained profoundly by the following reasons (Fig. 11)^41^:

1. The ionic radius of lead and cadmium ions has been determined 1.20 Å and 0.97 Å, respectively. Lead ions had both the highest size and sorption capability; hence, transferring the metallic ions on the HCP plane was more crucial in the adsorption capability relative to the microporous adsorption (which prefers smaller ions).
2. The unhydrated ions with the larger size had the lower charge agglomeration, the charge was more distributed, and a looser binding was generated between the metallic ions and water moieties.
3. The ions with the lesser size had more hydration enthalpy and lesser contact with the sorbent.

It has resulted that the overcoming separation mechanism of metallic ions was a connection of ions with the functional moieties on the resin surface rather than the transferring of particles inside the holes since micro-pores tend to attract the small-size particles.

The other case that can explain the mechanism is mass transfer. According to section “3.4”, the transfer of heavy metal ions into the pores was more dominant than the transfer of these metal ions onto the adsorbent interface. This case is owing to the higher value of diffusion coefficients relative to the mass transfer coefficients which relates to the convection mechanism (Table 7). Thus, Fick’s second law was used. The mass transfer parameters such as mass transfer coefficient and flux explained that each of these metal ions acted selectively in diffusing into the pores of the adsorbent. For instance, the K_L_ of lead and cadmium ions at a time of 45 min was calculated at 0.2028 × 10^15^ and 0.1054 × 10^15^ m/s, respectively, which expressed a higher tendency of lead ions for occupy the vacant sites than the cadmium ions. In other words, the concentration differential of heavy metal ions at various times created the driving force for the transfer of metallic ions from the solution into the pores of the adsorbent. It was essential to point this case that the transfer of lead and cadmium ions was associated with heat release because the enthalpy changes of lead and cadmium ions were determined as − 7.68 and − 6.62 kJ/mol , respectively. In addition, because the Gibbs changes (lead ions: − 0.7962 kJ/mol, cadmium ions: − 0.5408 kJ/mol) were lesser than 20 kJ/mol, the adsorption mechanism of lead and cadmium ions was physical which means that the generation of new materials or bonds has not occurred. In other words, the mobility of lead and cadmium ions into the channels of the adsorbent is only detected.

### Comparison

The comparison was conducted between the present adsorbent with the other hypercrosslinked adsorbent, and the results are displayed in Table 8. It could be concluded that the current sorbent except Chitosan/PVA had the biggest adsorption capability. Nevertheless, the porous hypercrosslinked adsorbent had a high uptake capacity in a binary system, but Chitosan/PVA had a high uptake capacity in a single-component system. Besides, chitosan was incorporated into PVA to promote the adsorption capability, but the current resin was not blended with the other components. Furthermore, the constructed resin had a high surface area. Hence, these data exhibited that the constructed resin was a crucial and eco-friendly sorbent for removing ions. Besides, a comparison was conducted between the present adsorbent and other diverse adsorbents in Table 9. Regarding this table, our hyper-cross-linked waste polycarbonate has a higher uptake capacity relative to the other adsorbents such as walnut shells, sulfonated magnetic nanoparticle adsorbent, inorganic oxide adsorbent, carbon aerogel, biochar, and kaolinite clay. It has resulted that cross-linking the waste polycarbonate is economical and also has excellent potential for adsorbing cadmium and lead ions from the wastewater. Indeed, the merits of the present work with the other adsorbents (Table 9) are disclosed as follows: (1) Converting the discarded materials into the applicable adsorbent which reduces the environmental pollution, (2) Decreasing the cost of the process because it doesn’t require to buy the monomer, and (3) According to Table 9, the hyper-cross-linked waste polycarbonate reveals a higher uptake capacity relative to the other adsorbents.

**Table 8:** Comparison hypercrosslinked polymers in removal of heavy metal ions.

Researcher	Precursor	Cross-linker	Surface area (m^2^/g)	Uptake Capacity (mg/g)			Ref
Liu et al	Crosslinked Chitosan/PVA	Ethylene Glycol Diglycidyl Ether	0.87	UO_2_			^42^
				156			
Monier et al	Crosslinked chitosan-2-aminopyridine	Glyoxal	56.8	Cu	Cd	Ni	^43^
				67	84	124	
Igberase et al	Crosslinked anionic polyelectrolytes	Glutaraldehyde	9.9	Cd			^44^
				0.373			
Zhang et al	Crosslinked polystyrene	Dimethyl Formamide	23.72	Cu	Pb	Hg	^45^
				1.1	1.3	0.8	
Akintola et al	Crosslinked polydithiocarbamates	Paraformaldehyde	11.50	Hg			^46^
				29.86			
Yu et al	Crosslinked chitosan coated with the maleic acid	Glutaraldehyde	19.161	Cd			^47^
				37.5			
Yang et al	Crosslinked chloromethylated poly(styrene-co-divinylbenzene)	1,2-Dichloroethane	167.98	Cd	Pb	Ni	^48^
				20	180	60	
Masoumi et al	Crosslinked waste polycarbonate	Formaldehyde Dimethyl Acetal	813.810	Cd	Pb		This work
				100	120

**Table 9:** Comparison the present adsorbent with the different adsorbents.

Researcher	Adsorbent	Heavy metal ion	Uptake capacity (mg/g)		Ref
Kamar et al	Walnut shells	Cd, Pb	Cd	Pb	^49^
			4.35	6.82	
Chen et al	Sulfonated magnetic nanoparticle adsorbent	Cd, Pb	Cd	Pb	^50^
			70	100	
Ciesielczyk et al	Inorganic oxide adsorbent	Cd, Pb	Cd	Pb	^51^
			94.05	102.02	
Kadirvelu et al	Carbon aerogel	Cd, Pb	Cd	Pb	^52^
			10	30	
Komkiene & Baltrenaite	Biochar	Cd, Pb	Cd	Pb	^53^
			3.50 μg/g	4.49 μg/g	
Adebowale et al	Kaolinite clay	Cd, Pb	Cd	Pb	^54^
			12	20	
Present adsorbent	Hyper-cross-linked waste polycarbonate	Cd, Pb	Cd	Pb	–
			100	120	

### Reusability test

**Figure 12:**
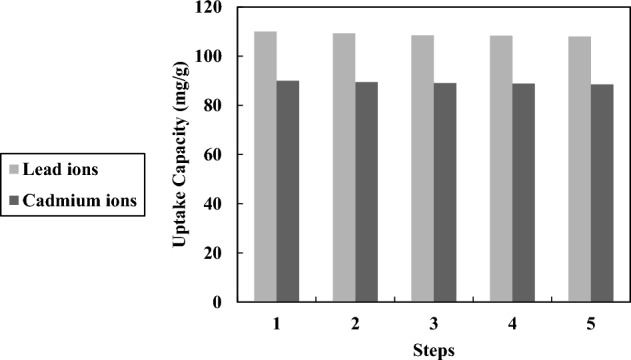
The reusability result for the hypercrosslinked polycarbonate.

The recyclability method of the hypercrosslinked waste polycarbonate is an important factor in industrial centers. For this purpose, 2 mol/L KCl was selected for leaching the cadmium and lead ions from the adsorbent. The results of the recyclability revealed that the adsorbent was suitable for extracting the metallic ions because the uptake capacity at the first and terminal points of lead ions has been found 110 mg/g and 108 mg/g, respectively (Fig. 12). In addition, the uptake capacity at the first and terminal points of cadmium ions has been found 90 mg/g and 88.50 mg/g, respectively (Fig. 12). Therefore, the hypercrosslinked polycarbonate is recommended to utilize more than five steps without reducing the uptake capacity, proving that the sorbent is completely recycled and used frequently.

## Conclusion

In this research, the waste polycarbonate was hypercrosslinked during the Friedel–Crafts reaction for the elimination of the lead and cadmium ions from the polluted solution. The synthesized polymeric resin has a high surface area and suitable pore volume for adsorption of the heavy metals. The adsorbent behavior for the metal removal was evaluated using isotherm models. The results showed that Freundlich and Toth were the best models for the cadmium and lead ions, respectively, and the adsorption process was multi-layer. The pseudo-second-order was the best model for kinetic modeling of the adsorption. In the thermodynamic aspect, the Gibbs energy changes for lead and cadmium ions were negative at entire temperatures except 80 °C which declared the adsorption is spontaneous. Also, the Gibbs energy changes tend to close to zero with temperature increasing, thus, temperature enhancement was not desired for the process. The reusability test showed that the adsorbent could be used frequently. In addition, the pH results showed that the stability of the adsorbent in acidic and basic conditions was suitable. The BET, TGA, and FTIR proved the adsorbent has many mesopores, good thermal stability, and the presence of many aromatic rings in the adsorbent structure.

## Data Availability

The datasets used and/or analyzed during the current study available from the corresponding author on reasonable request.
